# Effectiveness of the ChAdOx1 nCoV-19 Coronavirus Vaccine (Covishield^TM^) in Preventing SARS-CoV2 Infection, Chennai, Tamil Nadu, India, 2021

**DOI:** 10.3390/vaccines10060970

**Published:** 2022-06-17

**Authors:** Sharan Murali, Manikandanesan Sakthivel, Kamaraj Pattabi, Vettrichelvan Venkatasamy, Jeromie Wesley Vivian Thangaraj, Anita Shete, Alby John Varghese, Jaganathan Arjun, Chethrapilly Purushothaman Girish Kumar, Pragya D Yadav, Rima Sahay, Triparna Majumdar, Manisha Dudhmal, Azhagendran Sivalingam, Sudha Rani Dhanapal, Augustine Durai Samy, Vijayaprabha Radhakrishnan, Murali Mohan Muni Krishnaiah, Suresh Arunachalam, Punita Muni Krishna Gandhi, Elavarasu Govindasamy, Prabhakaran Chinnappan, Dhana Priya Vadhani Sekar, Prakash Marappan, Ezhil Pounraj, Parasuraman Ganeshkumar, Murugesan Jagadeesan, Manish Narnaware, Gagandeep Singh Bedi, Prabhdeep Kaur, Manoj Murhekar

**Affiliations:** 1 Indian Council of Medical Research—National Institute of Epidemiology, Tamil Nadu Housing Board, Chennai 600077, India; sharanmurali@nieicmr.org.in (S.M.); drnesan@nieicmr.org.in (M.S.); kamaraj@nie.gov.in (K.P.); vettrichelvan@nieicmr.org.in (V.V.); stanjeromie@nieicmr.org.in (J.W.V.T.); girishkumar@nie.gov.in (C.P.G.K.); azhagendrans@gmail.com (A.S.); sudharani.ph@gmail.com (S.R.D.); bhagust@gmail.com (A.D.S.); prabhasankar8@gmail.com (V.R.); muralimohan5764@gmail.com (M.M.M.K.); asuresh1819@gmail.com (S.A.); punithamkg@gmail.com (P.M.K.G.); elavarasug@yahoo.com (E.G.); cpraba1963@gmail.com (P.C.); vadhanisekar@gmail.com (D.P.V.S.); prakashmero88@gmail.com (P.M.); jezhil1987@gmail.com (E.P.); ganeshkumar@nieicmr.org.in (P.G.); mmurhekar@nieicmr.org.in (M.M.); 2 Indian Council of Medical Research—National Institute of Virology, Pune 411001, India; sheteaich.a-niv@gov.in (A.S.); yadav.pragya@gov.in (P.D.Y.); sahay.rr@gov.in (R.S.); triparna.majumdar@gmail.com (T.M.); dudhmalmanisha23@gmail.com (M.D.); 3 Greater Chennai Corporation, Ripon Building, Chennai 600003, India; collrpctlr@gmail.com (A.J.V.); drjaganathan@gmail.com (J.A.); ho@chennaicorporation.gov.in (M.J.); dchealth@chennaicorporation.gov.in (M.N.); commissioner@chennaicorporation.gov.in (G.S.B.)

**Keywords:** COVID-19 vaccines, vaccine effectiveness, ChAdOx1 nCoV-19, Covishield, AZD 1222, SARS-CoV-2 inactivated vaccines

## Abstract

We estimated the effectiveness of two doses of the ChAdOx1 nCoV-19 (Covishield) vaccine against any COVID-19 infection among individuals ≥45 years in Chennai, Tamil Nadu, India. A community-based cohort study was conducted from May to September 2021 in a selected geographic area in Chennai. The estimated sample size was 10,232. We enrolled 69,435 individuals, of which 21,793 were above 45 years. Two-dose coverage of Covishield in the 18+ and 45+ age group was 18% and 31%, respectively. Genomic analysis of 74 out of the 90 aliquots collected from the 303 COVID-19-positive individuals in the 45+ age group showed delta variants and their sub-lineages. The vaccine’s effectiveness against COVID-19 disease in the ≥45 age group was 61.3% (95% CI: 43.6–73.4) at least 2 weeks after receiving the second dose of Covishield. We demonstrated the effectiveness of two doses of the ChAdOx1 vaccine against the delta variant in the general population of Chennai. We recommend similar future studies considering emerging variants and newer vaccines. Two-dose vaccine coverage could be ensured to protect against COVID-19 infection.

## 1. Introduction

The World Health Organization (WHO) declared COVID-19 disease a pandemic in March 2020 [1]. India reported its first COVID-19 case on 30 January 2020 [2]. The first wave of the disease in India peaked in mid-September and declined gradually by October 2020. The country witnessed the second wave from February to July 2021, with new cases reaching over 400,000 per day [3]. COVID-19 deaths were reportedly higher among older adults and individuals with chronic morbid conditions [2]. Chennai, the capital city of Tamil Nadu state in India, was one of the hotspots. The delta variant of the SARS-CoV2 virus drove the second wave, which peaked in early May 2021 in Chennai, India.

The Government of India (GoI) approved two COVID-19 vaccines for public use in January 2021, namely Covishield and Covaxin [4]. Covishield is the brand name for the ChAdOx1 nCoV-19 Coronavirus vaccine (Recombinant) manufactured by the Serum Institute, Pune, India. ChAdOx1 is a chimpanzee adenovirus–vector vaccine with SARS-CoV2 spike protein, similar to the AZD1222 COVID-19 vaccine, manufactured by AstraZeneca [5]. The second vaccine, Covaxin (BBV152), is the whole virion inactivated SARS-CoV2 vaccine (BBV152) manufactured by Bharat Biotech in association with the Indian Council of Medical Research (ICMR)-National Institute of Virology (NIV) [6]. The Greater Chennai Corporation (GCC), the governing body of Chennai city, Tamil Nadu, India, included both vaccines in the COVID-19 vaccination drive.

India started COVID-19 vaccination on 1 March 2021, for individuals aged 60+ years and individuals with comorbidities in the 45+ age group. The government expanded the vaccine program in a phased manner to different age groups depending on the availability of vaccines and the health system’s preparedness. From 1 April 2021, India expanded vaccination to all individuals above 45 years and added 18–45-year-old individuals from 1 May 2021.

The second wave of COVID-19 began in India in February–March 2021. The majority of the COVID-19 infections (70%) in Tamil Nadu, India, between December and May 2021 were due to delta variants [7]. Pooled analysis from three randomized controlled trials conducted in the United Kingdom, Brazil, and South Africa documented the efficacy of ChAdOx1 nCoV-19 (AZD1222) at around 70% [8]. Few studies demonstrated the immune evasion property of the delta variant (B.1.617.2) and reduced effectiveness of vaccines [9,10,11]. Although there is ample evidence for the effectiveness of the COVID-19 vaccine in preventing severe diseases [12,13,14], there was limited data regarding its effectiveness in preventing COVID-19 infections.

In this context, a collaborative team from GCC and a public health research institution conducted a community-based study documenting the vaccine effectiveness (VE). Our primary objective was to estimate the effectiveness of two doses of the ChAdOx1 nCoV-19 (Covishield) vaccine against RT-PCR confirmed COVID-19 infection among the individuals ≥45 years during the second wave of COVID-19 driven by the delta variant, Chennai, India. We also determined the distribution of SARS-CoV-2 variants of concern (VoC) among a sample of individuals diagnosed with COVID-19.

## 2. Materials and Methods

### 2.1. Study Design and Setting

We conducted a community-based cohort study in Chennai from 22 May 2021, to 30 September 2021. Chennai is India’s fourth largest metropolitan city and the capital city of Tamil Nadu State, India. Greater Chennai Corporation (GCC) is divided into north, south, and central regions, with five zones each. Each zone is further divided into divisions, and there are 200 divisions. We reviewed the division-wise incidence of COVID-19 by month. Based on the previous four months of data, we identified divisions that showed a rising trend in COVID-19 incidence to ensure an adequate number of outcome events in the cohort (Supplementary Figure S1). Therefore, we selected the three divisions: 147, 151, and 153. We established the cohort in these divisions and collected data using the COVID-19 surveillance system established by GCC [15].

Chennai registered its first COVID-19 case on 14 March 2020. The city reported 566,147 cases of COVID-19 from the pandemic’s start till 31 December 2021. As part of the COVID-19 mitigation strategies, GCC has been conducting active surveillance, contact tracing, testing at community-level special camps, and follow-up of COVID-19-positive patients. For active door-to-door syndromic surveillance, GCC recruited temporary paid volunteers named Fever Survey Workers (FSWs), who visited every household twice a week to inquire about symptoms suggestive of COVID-19. They motivated individuals with at least one of the COVID-19 symptoms to get tested for COVID-19 free of cost at the nearest testing facility. High-risk contacts of the RT-PCR-positive COVID-19-infected patients were also tested, irrespective of the symptoms. In addition, symptomatic patients also sought care at the government or private health facilities. All health facilities across the city sent their samples for the RT-PCR tests for SARS-CoV-2 to the government-authorized laboratory. All laboratories uploaded the COVID-19 RT-PCR test results into a specially designed web portal named “igotit”, which generated line lists for every division.

FSWs collected the line list of RT-PCR-positive patients from the division office and contacted them. The division-level medical team triaged these individuals using a standardized protocol (Supplementary Figure S2). The FSWs also coordinated contact tracing and their testing. The medical team provided essential medication and counseling for patients in home isolation. The medical team referred the patients requiring hospitalization to designated COVID-19 hospitals based on the triaging. The telemedicine team at the zonal level monitored the patients for any red flag signs (persistent fever for more than five days or persistent cough or breathlessness, or SpO2 < 94%) daily and directed them to the nearest healthcare facility if needed (Supplementary Figure S2).

### 2.2. Study Population and Period

**Figure 1:**
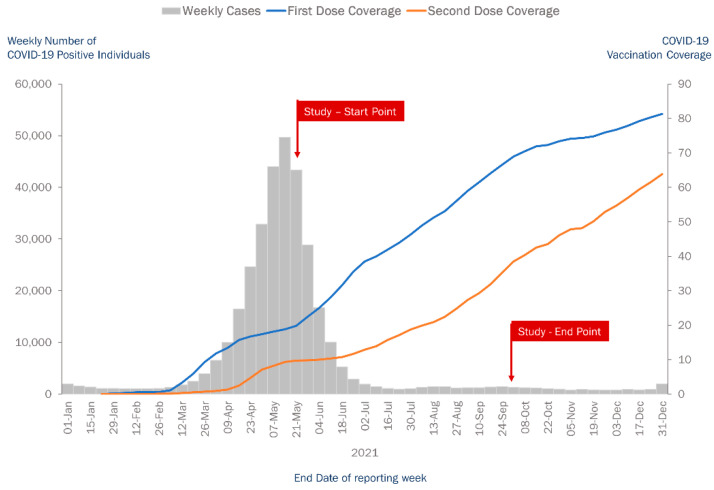
Vaccine coverage among the eligible population (18+) and the total number of reported COVID-19 cases by week, Chennai, Tamil Nadu, India, January–December 2021.

We conducted the study during the declining phase of the second wave (June–September 2021) of COVID-19 in Chennai City. The vaccination coverage among the eligible population (18+) was picking up rapidly (Figure 1). By May 22, 2021, about 20% of individuals ≥45 years received both doses of the COVID-19 vaccine.

Inclusion criteria: We included all individuals who gave consent in the study area. India opened the COVID-19 vaccination camp for the 18–45 age group from 1 May 2021. Hence, the coverage for this age group was low and limited to one dose only. Similarly, the coverage for Covaxin was only 12% in Chennai city. Therefore, we included only the 45+ age group for estimation of the vaccine effectiveness of Covishield.

Exclusion criteria: We excluded non-household settlements such as hostels, paying guest facilities, and residential hotels.

### 2.3. Sample Size and Sampling Strategy

We assumed 70% VE for the sample size calculation [8,16,17]. Based on the surveillance data for the 2 peak months in the first wave, we considered the attack rate among the unvaccinated group as 2%, confidence level of 95%, power of 80%, and desired precision width of 15%. The minimum required sample size was 13,467 individuals ≥45 years [18].

We enumerated all the households in the three divisions of Chennai city. We line listed all the individuals in the selected households. We applied the inclusion criteria mentioned above for data analysis.

### 2.4. Data Collection

We formed field teams consisting of supervisors with research experience and GCC Fever Survey Workers. The FSWs visited each household in the area assigned to them to assess their eligibility. After obtaining written informed consent from the eligible households, we assigned a unique identification number. We collected baseline data, including socio-demographic details, residential area details (slum/non-slum), and housing patterns (number of living rooms and total individuals in the house). We collected comorbidity status, history of previous COVID-19 infection, current COVID-19 disease, and vaccination details using an Open Data Kit Tool (ODK) [19]. We updated the exposure and outcome data weekly until 30 September 2021.

#### 2.4.1. Exposure Assessment

We confirmed the vaccination status by verifying the vaccination card given to the patient (based either on the message received in the beneficiary phone number or the vaccination certificate downloaded from the portal exclusively maintained by MoHFW for COVID-19 vaccination (COWIN).

We defined unvaccinated individuals as those who have not received the COVID-19 vaccine or were within 14 days of receiving the first dose [20]. We defined vaccination with a single dose as individuals who had completed 14 days after receiving the first dose of the vaccine and had not received the second dose [20,21]. The individuals who received the second dose but were within 14 days were also considered in this category. Vaccination with two doses included individuals who had completed 14 days after receiving the second dose of the vaccine.

#### 2.4.2. Outcome Measures

The primary outcome measure was RT-PCR-confirmed COVID-19 infection. We collected the line list of RT-PCR-positive individuals. Fourteen days after the confirmation of diagnosis, the data collectors telephonically interviewed the individuals to ascertain the clinical outcome of the COVID-19 infection as recovered or dead. Among the recovered, we defined severe disease as patients who required ventilator or oxygen support. We could not consider hospitalization a proxy for severe disease as there were no specific hospitalization criteria in the private sector. If a COVID-19-positive individual died within 28 days from the date of diagnosis of COVID-19 disease, we considered the event as a COVID death. However, death due to non-natural causes (e.g., accidental, intentional self-harm, and homicide) was not deemed a COVID-19 death even if it was within 28 days from the date of diagnosis of COVID-19 disease [22].

#### 2.4.3. Sample Collection and Laboratory Investigations

We collected nasal and oro-pharyngeal (N/OP) swabs from RT-PCR-confirmed COVID-19 patients aged above 45 years. The team shipped an aliquot of the COVID-19-positive samples to ICMR-National Institute of Virology, Pune, for next-generation sequencing (NGS).

Viral RNA was extracted (N/OP) from swab samples using an RNA extraction kit (Applied Biosystem, Waltham, MA, USA) and tested for the detection of the E gene using real-time RT-PCR [23]. We included samples with Ct < 30 for NGS. In brief, the COVIDSeq protocol (Illumina Inc., San Diego, CA, USA) includes amplification, tagmentation of the cDNA, and quantification of purified libraries using the KAPA Library Quantification Kit (Kapa Biosystems, Roche Diagnostics Corporation, Pleasanton, CA, USA). For sequencing, pooled libraries were denatured, neutralized, and loaded at a concentration of 1.4 pM onto the NextSeq 500/550 system using High Output Kit v2.5 (75 Cycles) [24]. The Bcl files generated were converted to fastq using bcl2fastq2 Conversion Software v2.20. We performed reference-based mapping using CLC Genomics Workbench v.22.0 to retrieve the sequence of SARS-CoV-2. We generated the phylogenetic tree using MEGA software version 10.

### 2.5. Statistical Analysis

We described the socio-demographic characteristics and vaccination profile of the individuals ≥45 years included in the cohort. We calculated the frequencies and proportions of the socio-demographics for the overall respondents and the 45–98 years age group. We summarized the vaccination profile by vaccination status, type of vaccine, and the number of doses for each age group (0–17, 18–44, 45–59, and 60–98 years) and gender as proportions.

**Figure 2:**
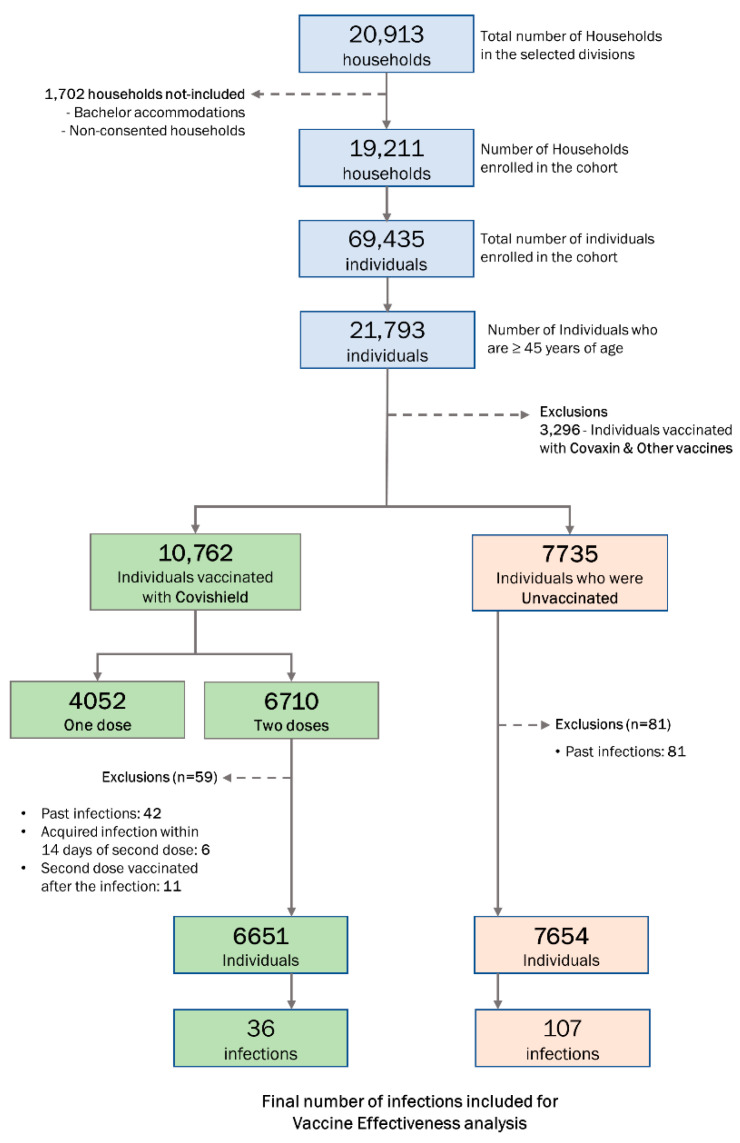
Participant enrollment, events, and data exclusions for estimating the effectiveness of ChAdOx1 nCoV-19 coronavirus vaccine (CovishieldTM) in reducing the delta variant of SARS-CoV2 infection, Chennai, Tamil Nadu, India, 2021.

We excluded individuals who took only one dose of Covishield or any other vaccine to estimate the vaccine effectiveness. We also excluded any COVID-19 individuals before the study’s initiation date (Figure 2).

We calculated the COVID-19 incidence per 100,000 population by age and gender for the individuals included in the analysis. We also summarized the COVID-19 disease outcome for the individuals who had COVID-19 infection during the study period as proportions.

We calculated the unadjusted relative risk (RR) and 95% confidence interval (95% CI) of COVID-19 infection for those who received two doses of the Covishield vaccine. We calculated the unadjusted vaccine effectiveness using the formula 1–RR and the 95% CI.

Further, we estimated the adjusted relative risk (ARR) with the 95% CI using Mantel–Haenszel stratified analysis for each socio-demographic characteristic, such as age, gender, comorbidity, residential area, and the number of individuals residing in a room. We also calculated the relative risk after adjusting for both age and gender. We used the relationship 1-ARR to calculate the strata-adjusted vaccine effectiveness with its 95% CI for two doses of Covishield against COVID-19 infection. We calculated power considering a 95% confidence level for two doses of Covishield in preventing infections and for severe disease. The data team used STATA SE (version 17.0) software (StataCorp LLC, College Station, TX, USA) for statistical analysis [25].

## 3. Results

### 3.1. Socio-Demographic Characteristics

Of the 20,913 households in the 3 divisions in Chennai, India, 19,211 (92%) were enrolled in the cohort (Figure 2). A total of 69,435 individuals were residing in the enrolled households. One-fifth (n = 3466; 18%) of the households were in slum areas, and 95.5% were pucca houses (Supplementary Table S1). Of the 69,435 individuals, nearly half (n = 34,500; 49.7%) were males, and 31.4% (n = 21,793) belonged to the 45+ age group (Supplementary Table S2). Over half (n = 35,152; 50.6%) had formal school education, and 39.4% (n = 27,365) had a college education. About 9% (n = 4912) of the individuals had a history of diabetes mellitus.

We included 21,793 individuals ≥45 years to analyze the vaccine effectiveness (Table 1). About 60% (n = 13,114) belonged to the 45–59 age group, and nearly half (n = 10,916; 50.1%) were males. The proportion of health care and frontline workers was 2.4% (n = 532).

### 3.2. Vaccination Status

Of the 21,793 individuals in the 45+ age cohort, 7735 (36%) were unvaccinated. About half (n = 10,762; 49.4%) of the 45+ cohort population received the Covishield vaccine (Table 2). The vaccination coverage was almost similar among males (n = 7191; 66%) and females (n = 6867; 63%). The coverage with at least one dose was highest (n = 5703; 65.7%) among the elderly (≥60 years), followed by the 45–59 age group (n = 8355; 63.7%).

### 3.3. COVID-19 Disease Outcomes

In the 45+ cohort of 21,793 individuals, 303 tested positive for COVID-19, with an incidence of 1390 per 100,000 population during the study period (Supplementary Table S3). Among the 14,305 individuals ≥45 years included in the primary analysis of VE, 143 tested positive for COVID-19 during the study period (Table 3). The incidence was higher among females (1034 per 100,000) than males (965 per 100,000). The incidence was higher in the 45–59 age group (1031 per 100,000) and the 60+ age group (956 per 100,000).

Among the 143 individuals who had COVID-19, 4.2% (n = 6) recovered from severe disease and 80.4% (n = 115) recovered without oxygen. The proportion of COVID-19 patients who had severe disease was 4.7% (n = 4) in the 45–59 age group and 3.5% (n = 2) in the 60+ age group (Table 3). Of the 5 (3.5%) COVID-19 deaths, 4 (7%) were in the ≥60 age group.

### 3.4. VE of Two Doses of Covishield

We excluded 3296 individuals vaccinated with Covaxin and vaccines other than Covishield from the primary analysis of vaccine effectiveness (VE). Among the 7735 unvaccinated individuals, we excluded 81 (1%) individuals who had past infections of COVID-19. Similarly, we excluded 59 (<1%) individuals from among the 6710 individuals vaccinated with two doses of the Covishield vaccine from the analysis for VE analysis (Figure 2).

The overall relative risk (RR) against COVID-19 infection at least 2 weeks after receiving two doses of the Covishield (ChAdOx1 nCoV-19) vaccine was estimated to be 0.39 (95% CI: 0.26–0.56). This translated to an overall vaccine effectiveness (VE) of 61.4% (95% CI: 43.6–73.6) (Table 4). The reported RR for the 45–59 age group was 0.34 (95% CI: 0.20–0.58) and that for the 60+ age group was 0.45 (95% CI: 0.26–0.78). The estimated VE was higher among the 45–59 age group (65.6% (95% CI: 42.3–79.5)) than 60+ age group (55.1% (95% CI: 21.7–74.2)).

We also calculated the RR and VE for the various subgroups within the 45+ cohort. After adjusting for the number of individuals per room in the household, the VE estimates showed a maximum of 63.8% (95% CI: 46.8–75.3). The covariate-adjusted VE estimates were almost closer to the overall VE reported in the study (Table 4). We estimated the age- and gender-adjusted VE as 61.6 % (95% CI: 43.8–73.7).

The power of the VE estimation for 2 doses of Covishield against any COVID-19 infection was 99.9%. However, the power for the VE estimate for 2 doses of Covishield against severe disease (oxygen/ventilator requirement) was only 34%. The estimated VE for a single dose of Covishield against COVID-19 infection in the 45+ age group was 28.7% (95% CI: −2.3–50.3; power: 45%).

### 3.5. Genomic Sequencing Results

We collected aliquots for 90 samples out of the 303 COVID-19 patients in the ≥45 age group from the respective testing laboratory. Out of 90 samples received at ICMR NIV Pune, only 75 (83%) samples had a CT value less than 30. We processed these 30 samples for NGS. The E gene copy numbers were found to be similar in the unvaccinated (7.7 × 10 1 to 2.1 × 108), one-dose (5.2 × 10 1 to 6.0 × 108), and two-dose (7.1 × 10 1 to 3.4 × 109) vaccinated groups.

**Figure 3:**
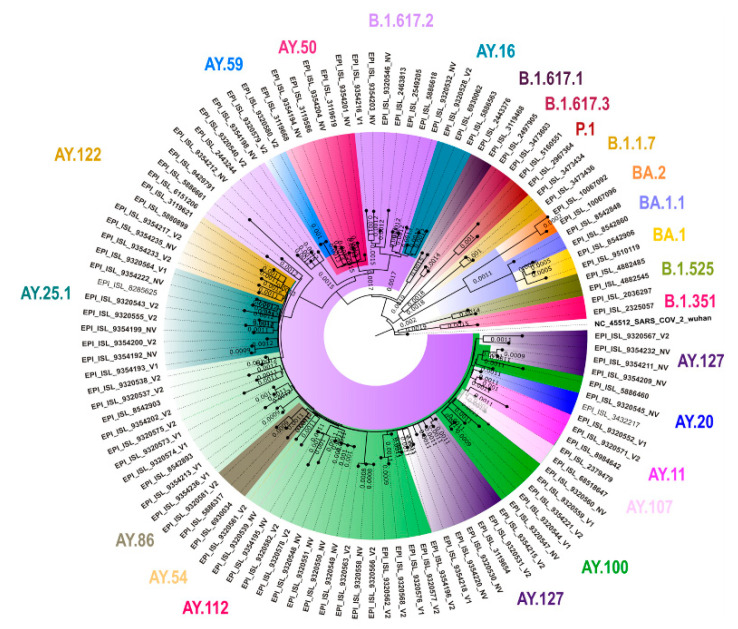
Neighbor joining tree of the SARS-CoV-2 sequences retrieved from the clinical samples collected from the 45+ age group individuals who were positive for SARS-CoV-2 in the COVID-19 vaccine effectiveness study, Chennai, India, 2021. We generated the tree using MEGA version 10 with the Tamura-3-parameter model with a gamma distribution as the rate parameter and bootstrap replication of 1000 cycles. The colored text represents the lineages. We visualized the generated tree using FigTree v1.4.4.

We retrieved genome > 98% from 67 samples. Therefore, we performed GSAID on these 67 samples only. All 67 samples tested positive for delta variants and sub-lineages, including AY. 100 (n = 16), B.1.617.2 (n = 14), AY.127 (n = 11), AY.25.1 (n = 8), AY.5.4 (n = 4) AY. 50 (n = 3), AY. 122 (n = 2), AY.59 (n = 2), and one each with AY.11, AY.16, AY.126, AY.20, AY.54, AY.107, and AY.86. We generated a neighbor joining tree using MEGA version 10 with the Tamura-3-parameter model with bootstrap replication of 1000 cycles (Figure 3).

Variant analysis in our study indicated the presence of the E484Q mutation in 2 out of 67 delta sub-lineages belonging to AY.100 (EPI_ISL_9320575_V2) and AY.25.1 (EPI_ISL_9354202_V2), respectively. Both patients were vaccinated with two doses of the Covishield vaccine. Two mutations of concern and interest in the RBD with evidence of increasing transmissibility or virulence, L452R, and T478K, were detected in 57 and 59 out of 67 samples, respectively. One of the mutations of concern in the furin cleavage site, P681R, was found in all sequences retrieved in the current study. All the above mutations were seen as a result of transversion of single nucleotide variants (SNVs) (Supplementary Figure S3).

## 4. Discussion

We conducted this study towards the latter half of the second wave, which overwhelmed the health system due to increased hospitalization. This study was initiated to generate locally relevant evidence to build trust in the effectiveness of the COVID-19 vaccine. Our study documented the vaccine effectiveness (VE) in a community cohort during the surge due to the delta variant in a metropolitan city in India. Two doses of Covishield effectively protected the population above 45 years from COVID-19 infection. This study included all sections of a population, hence generating data closer to the real-world setting. It was challenging to develop a community cohort in a pandemic setting due to the high burden on the health care workers in the field, apprehensions among the population, and poor linkages between disease and vaccination data. The available resources of GCC, trust among the public towards FSWs, and complementary expertise of the collaborators enabled the planning and execution of this study.

Our study reiterates the protective effect of vaccines against RT-PCR-confirmed COVID-19 infection as reported in three other studies from India. Our study’s VE estimates against COVID-19 disease were lower than those among a cohort of healthcare workers and comparable to two other studies from India [12,13,14]. The first study had unique characteristics, such as a large nationwide cohort that included relatively younger healthy HCWs and FLWs. The cohort size was 1.6 million, with and 82% 2-dose coverage of any COVID-19 vaccine and reported VE of 94.9% during January–May 2021 [12]. The second study, conducted from April to May 2021 using a test-negative case-control design among 4360 individuals (2379 cases and 1981 controls), estimated a 63% VE for 2 doses of Covishield in Delhi, India [14]. The third study was in a cohort of 10,567 health care workers, predominantly vaccinated with Covishield, and reported a VE of 65% from February to May 2021 in Tamil Nadu, India [13]. Irrespective of the study design or study population, the results consistently supported the usefulness of two doses of Covishield.

The VE in our study was also comparable to studies from other countries for the ChAdOx1 nCoV-19 (AZD1222) vaccine. The efficacy was 62% in a multi-centric trial across the UK, Brazil, and South Africa involving 11,636 participants for 2 doses of AZD1222 from April to November 2020 [26]. Subsequently, the surveillance data from the UK up to September 2021 documented a 60–70% VE against infection [27]. A meta-analysis of studies reporting the VE against delta estimated a pooled VE of 64% (57–71) after 2 doses of AZD1222 [28]. Given the evidence globally and from India, the vaccination program should focus on ensuring two-dose coverage of ChAdOx1 nCoV-19 (Covishield) to reduce the risk of COVID-19 infection.

Next-generation sequencing methods provide an added advantage in understanding the genetic epidemiology of the COVID-19-infected population. The present investigation revealed the predominance of delta and its sub-lineages during the study period. The variant analysis supported the transmissibility of the circulating delta lineages in the current study.

India’s vaccination program had two approved vaccines produced in India during the study period. The availability of manufacturing facilities in the country enabled rapid scaling of the vaccination program. A metanalysis pooled available data for multiple vaccines against delta variant infection and estimated the VE to be 84% for BNT162b2 (Pfizer–BioNTech) compared to 64% for AZD1222 (ChAdOx1 nCoV-19) [28]. Despite the lower VE of the AZD1222 vaccine, the availability at scale and operationalization of the vaccination program offered reasonable protection to the population against delt variant infection.

One of the limitations of the present study is that the data indicating the protection provided by the vaccine could have been influenced by natural immunity. A large proportion of SARS-CoV-2 infections are either asymptomatic or mildly symptomatic. Previous SARS-CoV-2 infection offers protection against subsequent infection [29,30]. Since the study was conducted after the initiation of the vaccination program, we did not test the participants to document their previous exposure to SARS-CoV-2 through serological assays. These assays do not differentiate antibodies on account of natural infection and vaccination. In the general population of Chennai, 2 serosurveys reported 30% and 43% as the seroprevalence for COVID-19 infection in October 2020 and January 2021, respectively [31,32,33]. Seroprevalence did not differ with age, gender, and socioeconomic groups. Hence, we assumed that the proportion of individuals seropositive for COVID-19 was similar in both vaccinated and unvaccinated groups.

The second limitation was the inability to include individuals with COVID-19 infections prior to the initiation of the study in the current analysis. The reasons for the lack of inclusion were the inability to ascertain the date of infection, the outcome of infection, and the accuracy of the interval between infection and vaccination.

The third limitation was an underestimation of symptomatic infections. We tried to overcome this limitation by using active surveillance for symptomatic infections and testing all symptomatic individuals and contacts. We pooled the line list of all COVID-19-positive individuals tested in any of the labs in the city to ensure completeness and representativeness. However, we might have missed individuals who did not reveal their symptom status and did not get tested.

Fourthly, a limitation was that the genome sequencing in the present study was conducted only in a sub-sample of the individuals diagnosed with COVID-19. Although the samples tested indicated a predominance of delta variants, a small proportion of infections might have been due to other variants. The type of variant might have influenced the outcomes for both the vaccinated and unvaccinated groups.

The next limitation was that both vaccinated and unvaccinated individuals might follow other practices for protection them from COVID-19 infections. We could not document this in the present study. However, a study from Chennai, December 2020, reported that only one out of three individuals wear masks appropriately outdoors [34].

Finally, the sample size was inadequate to study the VE against severe disease. Our primary objective was to understand the protective effect of COVID-19 vaccines irrespective of the severity of the disease in the general population. The number of individuals with severe diseases was inadequate for subgroup analysis. However, there is adequate evidence to support the usefulness of COVID-19 vaccines in preventing severe diseases in India. Two hospital-based case-control studies among RT-PCR-tested individuals reported around an 80% effectiveness for Covishield [14,35]. The effectiveness was 92–94% among a cohort of HCWs predominantly vaccinated with Covishield [13].

## 5. Conclusions

Our study findings support the hypothesis that two doses of the Covishield (ChAdOx1 nCoV-19) vaccine protected the adult population from COVID-19 infection in Chennai, India. We may require similar VE studies at the population level in the context of changing variants and newer vaccines and boosters. The disease surveillance (including hospitalization) and vaccination program databases are currently unlinked, limiting the opportunities for systematic periodic analysis of vaccine effectiveness. Effective linkages between databases help countries such as the United Kingdom report real-time vaccine effectiveness analyses [36]. We recommend linkages between the disease surveillance and vaccination databases at the national and state levels to enable regular, in-depth, real-time analysis of vaccine effectiveness. Our study supports that a high vaccination coverage with two doses should be ensured to maintain immunity against COVID-19 infection.

## Figures and Tables

**Table 1:** Socio-demographic characteristics of the individuals ≥45 years included in the analysis of the COVID-19 vaccine effectiveness cohort study, Chennai, 2021 (N = 21,793).

Characteristic		n	%
Division	147	8382	38.5
	151	4881	22.4
	153	8530	39.1
Gender	Male	10,916	50.1
	Female	10,844	49.8
	Transgender	33	0.1
Age group (in years)	45–59	13,114	60.2
	≥60	8679	39.8
Education	No formal education	2689	12.3
	School education	11,749	54.0
	College education	7355	33.7
Occupation	Health care/Frontline worker	532	2.4
	Others	21,261	97.6
Comorbidity (N = 21,788) *	Known HT	3770	17.3
	Known DM	4251	19.5
	Others	904	4.1
Vaccination status	Unvaccinated	7735	35.5
	Received only one dose	4972	22.8
	Received both the doses	9086	41.7
Person per room	<3	14,755	67.7
	≥3	7038	32.3
Residential Area	Slum	3271	15.0
	Non-Slum	18,522	85.0
HT—hypertension			
DM—diabetes mellitus			

* Missing for five individuals.

**Table 2:** Vaccination profile of the individuals ≥45 years in the COVID-19 vaccine effectiveness cohort study, Chennai, 2021 (N = 21,793) *.

Characteristic		N	Unvaccinated		Covishield				Covaxin				Other Vaccines	
					One Dose		Two Doses		One Dose		Two Doses			
			n	%	n	%	n	%	n	%	n	%	n	%
**Overall**	Overall	21,793	7735	35.5	4052	18.6	6710	30.8	904	4.1	2357	10.8	35	0.2
**Gender**	Male	10,916	3725	34.1	2024	18.5	3498	32.0	419	3.8	1228	11.2	22	0.2
	Female /TG	10,877	4010	36.9	2028	18.6	3212	29.5	485	4.5	1129	10.4	13	0.1
**Age Group** **(in years)**	45–59	13,114	4759	36.3	2828	21.6	3656	27.9	570	4.3	1278	9.7	23	0.2
	≥60	8679	2976	34.3	1224	14.1	3054	35.2	334	3.8	1079	12.4	12	0.1

* Individuals who received the vaccine after COVID-19 infection are also included here.

**Table 3:** COVID-19 incidence and its outcomes among the individuals ≥45 years in the COVID-19 vaccine effectiveness cohort study, Chennai, 2021 (N = 14,305).

Characteristic		Total	COVID-19 Positive	Incidence per 100,000 Population	Recovered without Oxygen		Severe Disease		Died		No Information	
		N			n	%	n	%	n	%	n	%
**Overall**		14,305	143	1000	115	80.4	6	4.2	5	3.5	17	11.9
**Gender**	Male	7150	69	965	58	84.1	2	2.9	2	2.9	7	10.1
	Female/TG	7155	74	1034	57	77.0	4	5.4	3	4.1	10	13.5
**Age Group** **(in years)**	45–59	8341	86	1031	69	80.2	4	4.7	1	1.2	12	14.0
	≥60	5964	57	956	46	80.7	2	3.5	4	7.0	5	8.8

**Table 4:** Effectiveness of two doses of the Covishield vaccine against COVID-19 infection among individuals ≥45 years, Chennai, India (N = 14,305).

Characteristics & Its Category		Vaccination Status	Total	COVID-19 Positive		RR (95% CI)	VE (95% CI)	Strata Adjusted RR * (95% CI)	Strata Adjusted VE * (95% CI)
			N	n	%				
**Overall**	**≥45 years**	Received two doses	6651	36	0.5	0.386 (0.264–0.564)	61.4 (43.6–73.6)		
		Unvaccinated	7654	107	1.4				
**Age Group** **(in years)**	**45–59**	Received two doses	3628	18	0.5	0.344 (0.205–0.577)	65.6 (42.3–79.5)		
		Unvaccinated	4713	68	1.4				
	**≥60**	Received two doses	3023	18	0.6	0.449 (0.258–0.783)	55.1 (21.7–74.2)		
		Unvaccinated	2941	39	1.3			0.386 (0.264–0.564)	61.4 (43.6–73.6)
**Gender**	**Male**	Received two doses	3463	18	0.5	0.376 (0.220–0.642)	62.4 (35.8–78.0)		
		Unvaccinated	3687	51	1.4				
	**Female/TG**	Received two doses	3188	18	0.6	0.400 (0.236–0.679)	60.0 (32.1–76.4)		
		Unvaccinated	3967	56	1.4			0.388 (0.266–0.565)	61.2 (43.5–73.4)
**Comorbidity**	**HT/DM**	Received two doses	1987	12	0.6	0.459 (0.231–0.916)	54.1 (8.4–77.0)		
		Unvaccinated	1826	24	1.3				
	**Non-HT/DM**	Received two doses	4663	24	0.5	0.361 (0.230–0.568)	63.9 (43.2–77.0)		
		Unvaccinated	5828	83	1.4			0.389 (0.266–0.567)	61.1 (43.3–73.4)
**Person per room**	**<3**	Received two doses	5034	31	0.6	0.389 (0.256–0.591)	61.1 (40.9–74.4)		
		Unvaccinated	4610	73	1.6				
	**≥3**	Received two doses	1617	5	0.3	0.277 (0.108–0.706)	72.3 (29.4–89.2)		
		Unvaccinated	3044	34	1.1			0.362 (0.247–0.532)	63.8 (46.8–75.3)
**Residential Area**	**Slum**	Received two doses	528	0	0.0	0.091 (0.005–1.512)	90.9 (-51.2–99.5)		
		Unvaccinated	1683	18	1.1				
	**Non-Slum**	Received two doses	6123	36	0.6	0.394 (0.268–0.580)	60.6 (42.0–73.2)		
		Unvaccinated	5971	89	1.5			0.369 (0.251–0.541)	63.1 (45.9–74.9)

**Footnotes:** * Adjusted for individual strata using Mantel–Haenszel stratified analysis. 1 VE—vaccine effectiveness | CI—confidence interval | RR—relative risk. 2 Individuals who were unvaccinated and vaccinated with two doses of Covishield only were included in this analysis (n = 14,305). In total, 123 individuals who reported past infection, 6 individuals who acquired infection within 14 days of the second dose of Covishield, and 11 who were vaccinated with a second dose after the infection were excluded (n = 140).

## Data Availability

The data presented in this study are available on request from the corresponding author. The data are not publicly available due to ethical reasons.

## Supplementary Materials

The following supporting information can be downloaded at: https://www.mdpi.com/article/10.3390/vaccines10060970/s1, Figure S1: The trend in COVID-19 cases in the contiguous divisions of Greater Chennai Corporation, March 2020–April 2021; Figure S2: Tamil Nadu COVID-19 Case Management Protocol; Figure S3: Single nucleotide variations in the SARS CoV-2 sequences retrieved from the clinical samples collected from the 45+ age group individuals who were positive for SARS-CoV-2 in COVID-19 Vaccine effectiveness Study, Chennai, India, 2021; Table S1: Socio-demographic characteristics of the households participated in the COVID-19 vaccine effectiveness cohort study, Chennai, 2021 (N = 19,211); Table S2: Socio-demographic characteristics of the individuals who participated in the COVID-19 vaccine effectiveness cohort study, Chennai, 2021 (N = 69,435); Table S3: COVID-19 incidence and its outcomes among individuals ≥45 years in the COVID-19 vaccine effectiveness cohort study, Chennai, 2021 (N = 21,793).
