# Supplementary material for: Effectiveness of the ChAdOx1 nCoV-19 Coronavirus Vaccine (CovishieldTM) in Preventing SARS-CoV2 Infection, Chennai, Tamil Nadu, India, 2021
Source: Vaccines (Basel). 2022 Jun 17;10(6):970. doi: 10.3390/vaccines10060970 (PMC9228854; doi:10.3390/vaccines10060970)
Supplement: Supplementary file 1 [file vaccines-10-00970-s001.zip › vaccines-1731407-supplementary.pdf]

Effectiveness of ChAdOx1 nCoV-19 Corona Virus Vaccine (Covishield™) in preventing SARS-CoV2 infection, Chennai, Tamil Nadu, India, 2021

SUPPLEMENTARY FILE

**TABLES**	
S1	Socio-demographic characteristics of the households participated in the COVID-19 vaccine effectiveness cohort study, Chennai, 2021 (N=19,211)
S2	Socio-demographic characteristics of the individuals who participated in the COVID-19 vaccine effectiveness cohort study, Chennai, 2021 (N=69,435)
S3	COVID-19 incidence and its outcomes among individuals ≥45 years in the COVID-19 vaccine effectiveness cohort study, Chennai, 2021 (N=21,793)
**FIGURES**	
S1	The trend in COVID-19 cases in the contiguous divisions of Greater Chennai Corporation, March 2020 – April 2021
S2	Tamil Nadu COVID-19 Case Management Protocol
S3	Single nucleotide variations in the SARS CoV-2 sequences retrieved from the clinical samples collected from the 45+ age group individuals who were positive for SARS-CoV-2 in COVID-19 Vaccine effectiveness Study, Chennai, India, 2021

Supplementary Table S1. Socio-demographic characteristics of the households participated in the COVID-19 vaccine effectiveness cohort study, Chennai, 2021 (N=19,211)

Characteristic		n	%
Division	147	7,555	39.3
	151	4,155	21.6
	153	7,501	39.1
Area	Slum	3,466	18.0
	Non-Slum	15,745	82.0
Type of house	Kucha	197	1.0
	Semi pucca	658	3.4
	Pucca	18,356	95.5
Ration card (indicative of socio-economic status)	Below poverty line (BPL)	1,101	5.7
	Non-BPL	15,086	78.5
	No card	3,024	15.7
Number of family members	<4	8,831	46.0
	≥4	10,380	54.0
Rooms for sleeping	1	6,312	32.9
	2	10,113	52.6
	≥3	2,786	14.5
Number of people per room	1	2,765	14.4
	2	10,324	53.7
	3	4,069	21.2
	≥4	2,053	10.7

Supplementary Table S2. Socio-demographic characteristics of the individuals who participated in the COVID-19 vaccine effectiveness cohort study, Chennai, 2021 (N=69,435)

Characteristic		n	%
Division	147	27,760	40.0
	151	15,090	21.7
	153	26,585	38.3
Gender	Male	34,500	49.7
	Female	34,836	50.2
	Transgender	99	0.1
Age group (in years)	0 - 17	15,135	21.8
	18 - 44	32,507	46.8
	45 - 59	13,114	18.9
	60 - 98	8679	12.5
Education	No formal education	6918	10.0
	School education	35,152	50.6
	College education	27,365	39.4
Occupation	Health care / Frontline worker	1418	2.0
	Others	68,017	98.0
Comorbidity	Known HT	4283	7.9
	Known DM	4912	9.0
	Others	1138	2.1
Vaccination status	Unvaccinated	38,892	56.0
	Received only one dose	16,700	24.1
	Received both the doses	13,843	19.9

HT - Hypertension

DM - Diabetes Mellitus

Supplementary Table S3. COVID-19 incidence and its outcomes among the individuals ≥45 years in the COVID-19 vaccine effectiveness cohort study, Chennai, 2021 (N=21,793)

		Total	COVID-19 positive	Incidence per 100,000 population	Recovered without oxygen support		Recovered with Oxygen Support		Recovered with ventilator support		Died		No Information	
Characteristic			N		n	%	n	%	n	%	n	%	n	%
Overall		21,793	303	1390	259	85.5	20	6.6	1	0.3	12	4.0	11	3.6
Gender	Male	10,916	145	1328	123	84.8	8	5.5	0	0.0	7	4.8	7	4.8
	Female / TG	10,877	158	1453	136	86.1	12	7.6	1	0.6	5	3.2	4	2.5
Age Group (in years)	45 - 59	13,114	185	1411	167	90.3	9	4.9	1	0.5	1	0.5	7	3.8
	≥60	8679	118	1360	92	78.0	11	9.3	0	0.0	11	9.3	4	3.4

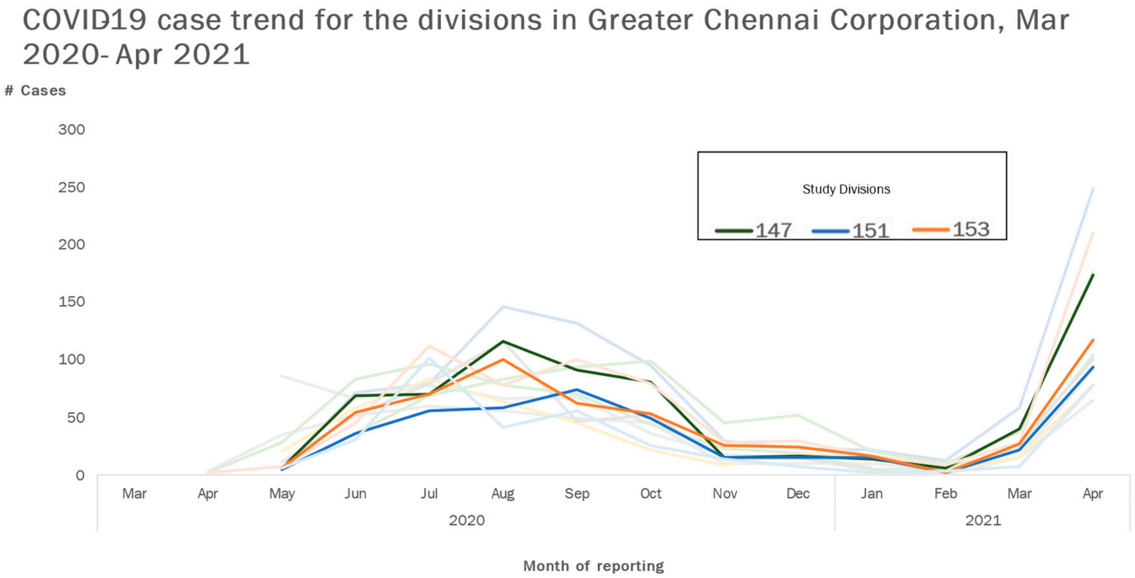

Supplementary Figure S1. The trend in COVID-19 cases in the contiguous divisions of Greater Chennai Corporation, March 2020–April 2021. The graph shows the rising trend in cases in the selected divisions 147, 151 and 153.

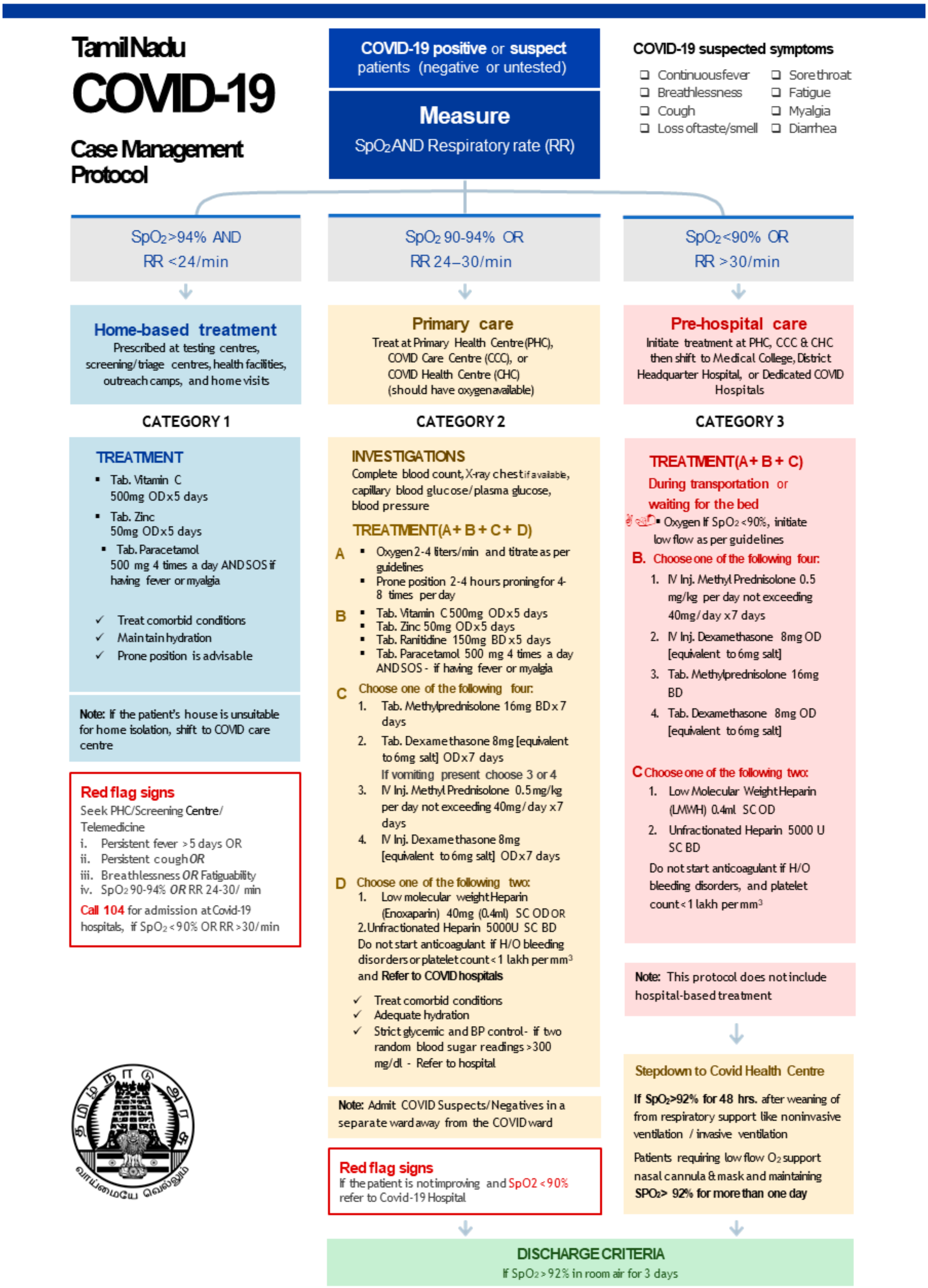

Supplementary Figure S2. Tamil Nadu COVID-19 Case Management Protocol

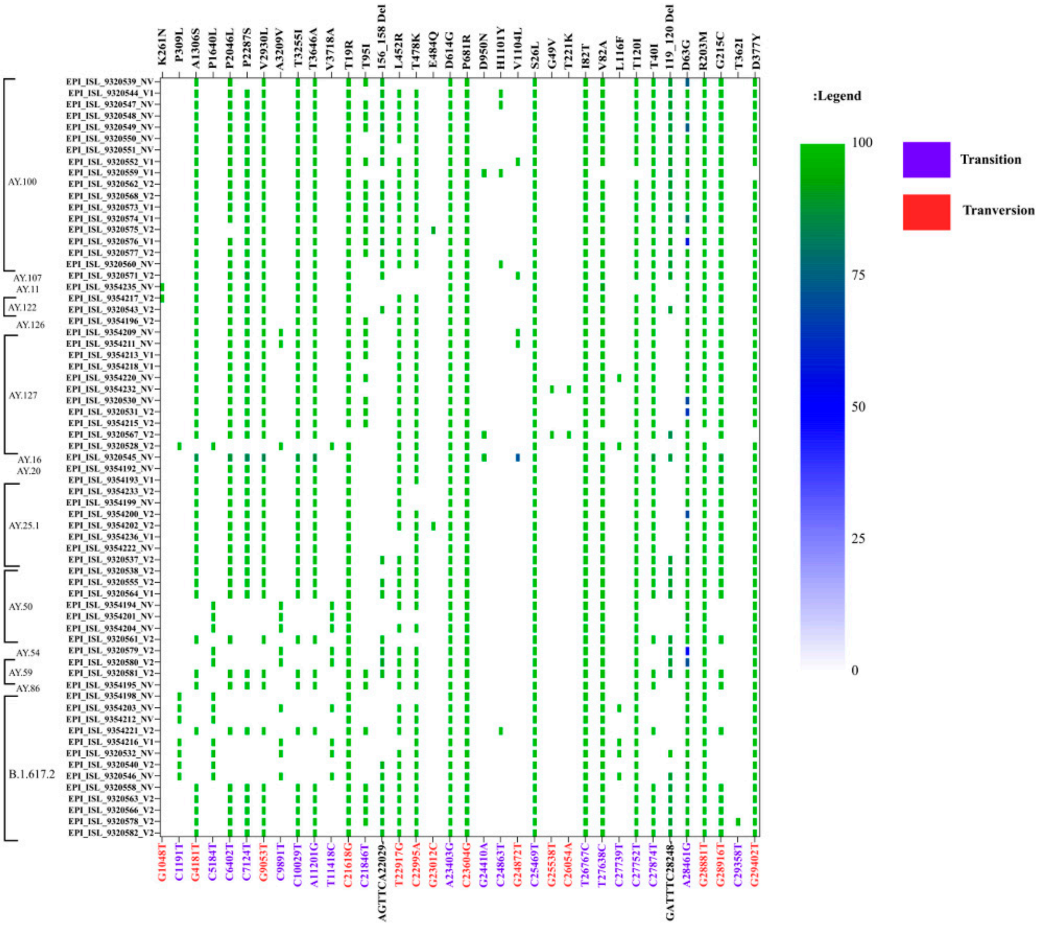

Supplementary Figure S3. Single nucleotide variations in the SARS CoV-2 sequences retrieved from the clinical samples collected from the 45+ age group individuals who were positive for SARS-CoV-2 in COVID-19 Vaccine effectiveness Study, Chennai, India, 2021.

7
